# Gut virome and metabolic associations in patients with acute pancreatitis

**DOI:** 10.1128/msystems.01400-25

**Published:** 2026-03-06

**Authors:** Min Liu, Lvyue Wang, Jianjun Liu, Qi Yuan, Yuetong Zhang, Siyu Wu, Yue Zhang, Ruochun Guo, Yidi Zhang, Tong Lu, Qiulong Yan, Shenghui Li, Guorui Xing, Bo Dong, Ning Zheng

**Affiliations:** 1 Department of Critical Care Medicine, The Second Hospital of Nanjing, Affiliated to Nanjing University of Chinese Medicine 531909, Nanjing, China; 2 Clinical Laboratory of Integrative Medicine, The First Affiliated Hospital of Dalian Medical University 74710, Dalian, China; 3 Puensum Genetech Institute, Wuhan, China; 4 Agro-biological Gene Research Center of Guangdong Academy of Agricultural Sciences, State Key Laboratory of Swine and Poultry Breeding Industry, Guangzhou, China; 5 Center for Microbiome Medicine, The Fifth Affiliated Hospital of Southern Medical University 604348, Guangzhou, China; Det Kongelige Akademi-Konservering, Copenhagen, Denmark

**Keywords:** gut virome, acute pancreatitis, biomarker, bacterial-viral-metabolite networks, metabolomics

## Abstract

Acute pancreatitis (AP) is a frequent inflammatory disorder with outcomes ranging from mild disease to severe forms marked by infection and organ failure. Gut microenvironment disruption and barrier dysfunction are increasingly recognized as key drivers of AP progression, yet most microbiome studies have focused on bacteria. The gut virome modulates bacterial ecology and host immune responses and remains poorly characterized in AP. We aimed to comprehensively profile virome alterations in AP and evaluate their associations with disease severity, etiology, and clinical parameters. Metagenomic sequencing data from AP patients and healthy controls (HCs) were analyzed using the viromic tools. Viral diversity, taxonomy, functional composition, and predicted viral-host linkages were profiled. Microbial-viral-metabolite networks were constructed, and classification performance was evaluated using random forest models. AP viromes exhibited significantly reduced Shannon and Simpson diversity and distinct β-diversity separation from HCs. AP-enriched phages predominantly targeted Parabacteroides, Escherichia, and Bacteroides, while HC-enriched phages were linked to SCFA-producing commensals. Functional analysis revealed enrichment of replication- and lysis-related auxiliary metabolic genes (AMGs) in AP-enriched viral operational taxonomic units (vOTUs), whereas HC-associated vOTUs carried stability-related functions. Severity- and etiology-stratified analyses indicated consistent enrichment of *Peduoviridae* infecting Enterobacteriaceae and higher prevalence of eukaryotic viruses in advanced stages. Network analyses revealed denser microbial-viral-metabolite interactions in AP, correlated with hepatobiliary and lipid metabolic markers. A minimal seven-virus panel achieved an AUC of 97.5% for AP classification. AP is characterized by profound gut virome remodeling reflecting disease severity and etiology, with diagnostic and mechanistic relevance for future therapeutic strategies.

**IMPORTANCE:**

This study highlights the gut virome as a previously underappreciated component of acute pancreatitis (AP)-associated dysbiosis and suggests that viral communities may influence disease severity and metabolic disturbances beyond bacterial effects alone. By demonstrating the diagnostic potential of virome-based signatures, our findings support expanding microbiome research in AP to include viral components, with implications for improved disease stratification and future therapeutic development.

## INTRODUCTION

Acute pancreatitis (AP) is an inflammatory disorder of the pancreas characterized by edema, hemorrhage, and necrosis, resulting from the autodigestion of pancreatic tissue triggered by diverse etiological factors. Clinically, it presents with acute epigastric pain and elevated serum amylase or lipase levels (1, 2). The global incidence of AP has risen in recent years, with approximately 20% of patients developing severe acute pancreatitis (SAP), which carries a mortality rate of 15%–30% (3–5). SAP often leads to serious complications, including pancreatic infection, sepsis, and multiple organ failure, placing a significant burden on patients and healthcare systems. In the United States alone, annual medical expenses associated with AP exceed $2.5 billion (6, 7). Understanding the underlying pathogenesis and identifying novel therapeutic targets is therefore of great importance.

Traditionally, AP has been attributed to factors, such as alcohol abuse and gallstones, accounting for 70%–80% of cases (8). Pathophysiological studies have focused primarily on premature activation of pancreatic enzymes and excessive systemic inflammation (9). However, emerging research highlights the crucial role of gut microenvironment dysregulation in AP onset and progression (10). The gut microbiome—a dynamic ecosystem comprising bacteria, fungi, viruses, and archaea—plays a central role in maintaining intestinal homeostasis, modulating immunity, and influencing host metabolism (11). Among these components, bacterial dysbiosis has been most extensively studied. For example, AP patients, particularly those with SAP, display a notable decrease in beneficial bacteria (e.g., *Bifidobacterium*, *Lactobacillus*) and an overgrowth of opportunistic pathogens, such as *Escherichia coli* and *Enterococcus* spp. (12–15). These alterations are associated with disease severity and may amplify systemic inflammation and pancreatic injury.

While bacterial dysbiosis has garnered significant attention, the gut virome—comprising bacteriophages, eukaryotic viruses, and viral nucleic acids derived from lysed particles or cells (16)—may serve as a previously underappreciated but crucial modulator in AP pathogenesis. For instance, in inflammatory bowel disease (IBD), virome diversity is reduced, and temperate phages undergo lysogenic-to-lytic transitions that can exacerbate inflammation (17). It is important to note, however, that the relationship is complex. A recent methodological study of unamplified viromes found that global α- and β-diversity metrics alone were not efficient discriminators of IBD status, highlighting the high interpersonal variability and temporal dynamics of the virome (18). Similar virome perturbations have been observed in metabolic disorders (e.g., obesity, type 2 diabetes) and autoimmune or neurodevelopmental conditions (16, 19–21). Despite this growing recognition, the role of the gut virome in AP remains largely unexplored.

To address this knowledge gap, we performed an integrative metagenomic analysis using publicly available data sets. We investigated the compositional and functional differences in the gut virome between AP patients and healthy controls (HCs) and examined their associations with bacterial communities and serological indicators. This study aims to elucidate the virome’s role in AP pathogenesis and identify disease-associated viral signatures that could inform early diagnosis, patient stratification, and virome-targeted therapeutic strategies.

## MATERIALS AND METHODS

### Sample information

This study analyzed fecal samples from a total of 197 participants, including 82 patients diagnosed with AP and 115 healthy individuals, all from China. Among the AP patients, 19 were classified as having SAP, 25 as moderately severe AP (MSAP), and 38 as mild AP (MAP), based on disease severity. In terms of etiology, 51 patients were diagnosed with acute biliary pancreatitis (ABP), 12 with acute hyperlipidemic pancreatitis (AHP), 7 with tumor-related acute pancreatitis (APN), and the remaining 12 cases were attributed to other causes (Table S1). The data set was derived from the study by Liu et al. (22). Publicly available fecal metagenomic samples were sourced from the European Bioinformatics Institute, with the accession number PRJEB36300.

### Data preprocessing

Raw sequencing data were quality-controlled using fastp v0.20.1 (parameters: -l 90 -q 20 -u 30 -y --trim_poly_g) to remove low-quality reads. Subsequently, Bowtie2 v2.4.1 was employed to align reads to the GRCh38 reference genome, and host-derived sequences were filtered out (using default parameters) (23). Ultimately, after quality control and host read removal, each sample retained on average 13.7 ± 3.7 million reads, corresponding to 2.05 ± 0.55 Gb of high-quality non-host metagenomic data per sample.

### Gut virome profiling

The gut virome was characterized using the Chinese Gut Viral Catalog (cnGVC) (24), a comprehensive database constructed from over 10,000 metagenomes and comprising 93,462 non-redundant viral operational taxonomic units (vOTUs). Cleaned metagenomic reads were aligned to the cnGVC using Bowtie2. These vOTUs were pre-clustered in the cnGVC at ≥95% average nucleotide identity, representing species-level viral units. Cleaned metagenomic reads were aligned to these predefined vOTUs using Bowtie2 (25), and read counts were used to quantify vOTU abundance. To ensure accurate and non-redundant assignment, Bowtie2 was configured to report only the best alignment for each read. When multiple alignments received identical scores, the internal scoring scheme assigned the read to a single best hit. A vOTU was considered present in a sample only if the mapped reads covered at least 10% of the viral genome length. Relative abundances were then normalized by the total number of uniquely mapped reads per sample. For each vOTU, read counts were normalized by genome length (reads per kilobase), and these values were further scaled by the total number of mapped reads per sample to obtain length-adjusted relative abundances. Viral family-level abundances were obtained by aggregating vOTUs assigned to the same family. For taxonomic assignment, we adopted the most recent viral classification standards published by the International Committee on Taxonomy of Viruses (ICTV, https://ictv.global/).

### Gut virome functional profiling and host prediction

Functional and host-associated annotations of vOTUs were retrieved directly from the cnGVC catalog (24), which provides standardized and uniformly processed metadata for all viral genomes. In brief, the cnGVC pipeline performs ORF prediction using Prodigal v2.6.3 (26), annotates viral proteins against the Kyoto Encyclopedia of Genes and Genomes (KEGG) database to identify viral auxiliary metabolic genes (AMGs), and infers putative hosts using both CRISPR spacer matching and homology-based approaches. For KEGG-based AMG annotation, viral proteins were assigned KEGG orthologs (KOs) based on best-hit matches using DIAMOND with the parameters “-e 1e-5 –query-cover 50 –subject-cover 50 –min-score 50,” and KEGG-annotated proteins were treated as putative AMGs for downstream analyses. CRISPR-based host assignments were derived from spacer-vOTU alignments identified by BLASTn (bit-score ≥45 and full-length coverage). Homology-based host predictions were determined from BLASTn alignments between vOTUs and prokaryotic genomes with ≥90% nucleotide identity across ≥30% of the vOTU length. These curated annotations were used directly for downstream functional and host-association analyses in this study.

### Prediction of viral lifestyle

To infer the lifestyle of bacteriophages, viral contigs were analyzed using BacPHLIP (27), a machine-learning-based tool that predicts phage lifestyle (lytic or temperate) based on conserved protein domain content. To ensure reliable predictions, only viral genomes with an estimated completeness greater than 90%, as assessed during viral genome quality control, were included in this analysis. BacPHLIP was run with default parameters, and phages were classified as temperate or lytic according to the predicted lifestyle scores. Viral contigs that did not meet the completeness threshold were excluded to minimize uncertainty associated with fragmented genomes.

### Gut bacteriome profiling

The composition of the gut bacteriome was profiled from fecal metagenomic data using the MetaPhlAn4 tool (28), which enables clade-specific marker-based taxonomic profiling. Species-level relative abundances were first normalized within each sample. Higher-level taxonomic abundances (genus and phylum) were then derived by summing the relative abundances of all species belonging to the respective taxonomic ranks.

### Serum metabolome and lipidome analyses

The serum samples were prepared from whole blood by centrifugation at 1,500 × *g* for 10 min at 4°C, aliquoted, and stored at −80°C until analysis. Sample pretreatment was performed as follows: methanol (Fisher Scientific, Fair Lawn, USA) was added to 100 μL of serum, and the mixture was vortexed for 180 s. Subsequently, 900 μL of methyl tert-butyl ether (Sigma-Aldrich, St. Louis, USA) and 250 μL of Milli-Q (Merck KGaA, Darmstadt, Germany) purified water were added and vortexed for an additional 180 s. The mixture was incubated on a rolling mixer for 10 min and then kept at room temperature for 10 min, followed by centrifugation at 13,000 × *g* for 10 min at 4°C. Next, 700 μL of the lipid extract from the upper phase and 400 μL of the polar metabolite extract from the lower phase were transferred into two EP tubes and dried to completeness by vacuum centrifugation. The remaining samples were pooled, centrifuged, and similarly separated into upper and lower phases to generate quality control samples. Polar metabolite extracts were analyzed using three different analytical methods and were separated by reverse-phase chromatography with detection in positive and negative ionization modes, respectively. Lipid extracts were also separated chromatographically and analyzed in both positive and negative ionization modes.

Untargeted metabolome and lipidome quantification was performed using an Ultimate 3000 ultra-high-performance liquid chromatograph coupled to a Q Exactive quadrupole-Orbitrap high-resolution mass spectrometer (Thermo Scientific, USA). The chromatographic separation and mass spectrometry conditions for polar metabolites and lipids have been described in detail in our previous article. Polar metabolites were structurally annotated by searching acquired MS/MS spectra against a local in-house MS/MS library generated from authentic standards, the NIST 17 Tandem MS/MS library (National Institute of Standards and Technology), a local version of MoNA (MassBank of North America), and the mzCloud library (Thermo Scientific, USA). Untargeted lipidomics data were processed using LipidSearch software, including peak picking and lipid identification. Mass tolerances for precursor and product ion searches were set to ±5 ppm and 5 mDa, respectively, and the MS/MS similarity score threshold was set at 5.

### Statistical analysis

All statistical analyses and data visualization for this study were conducted in the R v4.0.3 environment. First, we calculated alpha diversity metrics (including observed vOTUs, Shannon index, and Simpson index) using the *vegan* package (v2.6-4), and used the vegdist function based on Bray–Curtis distance to perform principal coordinates analysis (PCoA) and PERMANOVA test to assess between-group differences. Pairwise PERMANOVA was conducted to assess beta-diversity differences between specific groups. For each pairwise comparison, Bray–Curtis distances were calculated from the species abundance matrix, and PERMANOVA was performed using the adonis2 function from the vegan package with 9,999 permutations. For biomarker identification, the Wilcoxon rank-sum test was used to compare vOTU abundances between groups. To account for potential confounding factors, multivariable association analysis was additionally performed using MaAsLin2, fitting generalized linear models with appropriate normalization and transformation as implemented in MaAsLin2. *P* values across cohorts were combined and subsequently adjusted for multiple testing using the Benjamini-Hochberg false discovery rate (FDR) procedure, with FDR-adjusted *P* < 0.05 considered statistically significant. In functional analysis, we calculated the distribution frequency of KO genes and evaluated inter-group differences using Fisher’s exact test. For machine learning modeling, the Random Forest algorithm was employed, followed by fivefold cross-validation with five repetitions, and the AUC values were computed using the pROC package to evaluate model performance, and cross-cohort validation was performed to test model robustness. Finally, Spearman correlation analysis was used to explore associations between viral and bacterial biomarkers and visualized using ggplot2.

## RESULTS

### Metagenomic data

In our study, we collected fecal samples from 82 AP patients and 115 HC subjects. Further analysis of the cohort revealed that the mean age of AP patients (49.5 ± 16.0) was significantly lower than that of HC (57.4 ± 7.6, *P* = 0.001, Table S1). The body mass index was also significantly higher in the AP group (24.4 ± 3.17) compared to the HC group (23.0 ± 2.52, *P* = 0.002). Notably, the proportion of female participants in the AP group was significantly higher (57.0%) compared to the HC group (43.0%, *P* = 0.001). Raw metagenomic samples were filtered to remove low-quality or human-derived reads, and the remaining clean reads from each sample were aligned to the cnGVC, with a total of 44,912 vOTUs that could be recovered.

### Diversity and structure of the AP gut virome and bacteriome

**Fig 1:**
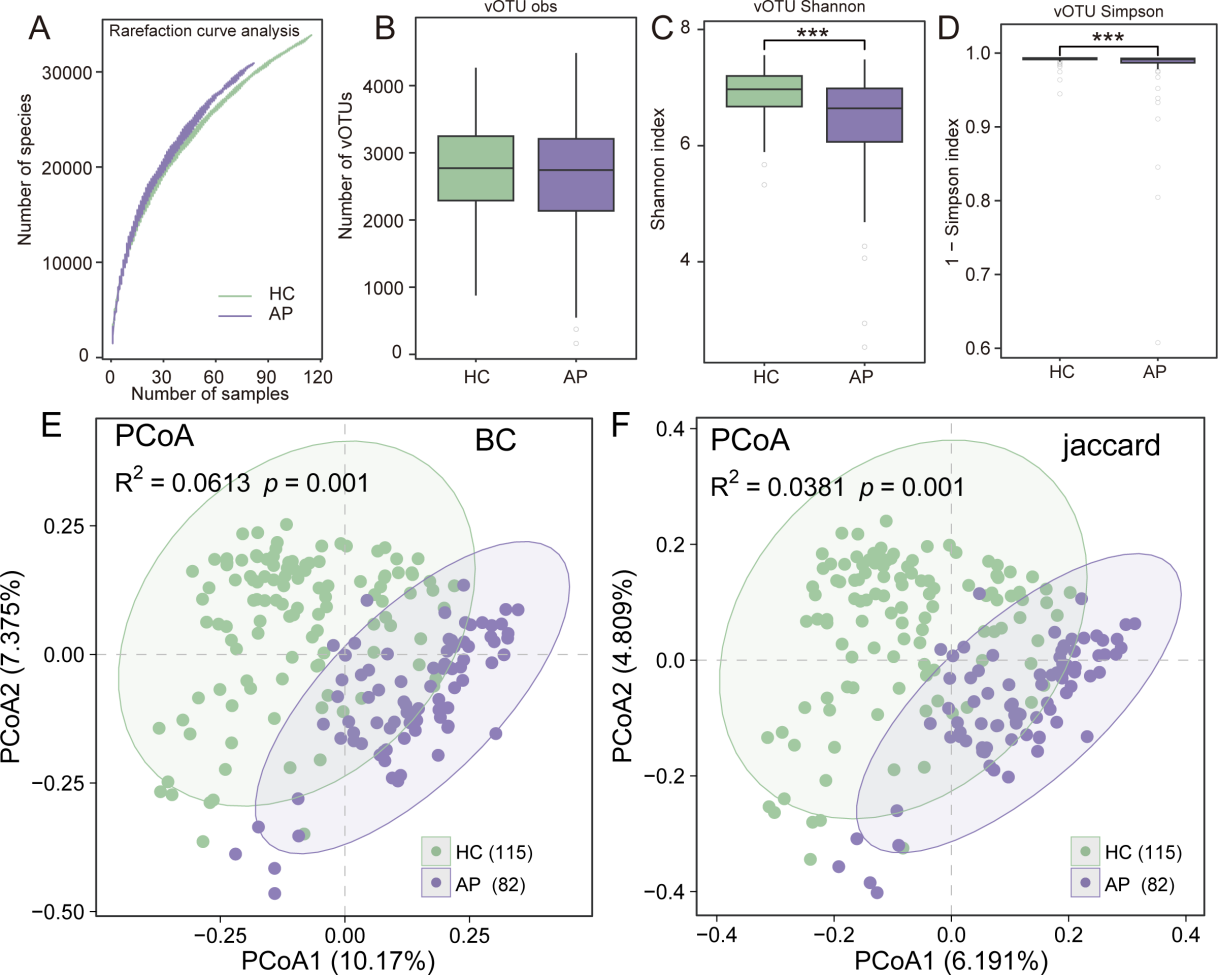
Diversity and compositional profiles of the gut virome in AP patients and HC groups. (**A**) Rarefaction curves showing the accumulation of observed vOTUs with increasing sample size in the AP and HC groups. (**B–D**) Boxplots displaying α-diversity metrics of the gut virome, including the observed number of vOTUs (**B**), Shannon index (**C**), and Simpson index (**D**), in AP patients and HCs. (**E**) PCoA of virome β-diversity based on Bray–Curtis dissimilarity. (**F**) PCoA of virome β-diversity based on Jaccard distance. Each point represents one sample, and ellipses indicate the 95% confidence intervals for each group.

Rarefaction curves revealed that although a substantial portion of viral diversity was captured, sequencing depth and sample size did not allow the curves to reach full saturation, suggesting that certain low-abundance vOTUs may have remained undetected (Fig. 1A). Although the observed number of vOTUs did not differ significantly between AP and HC groups (*P* > 0.05, Fig. 1B), both Shannon and Simpson diversity indices were significantly reduced in AP patients (*P* < 0.001, Fig. 1C and D), indicating a notable decline in viral community evenness. Beta diversity analysis based on Bray–Curtis dissimilarity revealed a significant difference in virome composition between AP and HC groups (R² = 0.0613, *P* = 0.001; Fig. 1E). PCoA of the Bray–Curtis distance matrix further illustrated this separation, with PCoA1 and PCoA2 explaining 10.17% and 7.38% of the total variance, respectively. Consistently, PCoA based on Jaccard distance, which emphasizes presence–absence patterns, also revealed a clear separation between the two groups (R² = 0.0381, *P* = 0.001; Fig. 1F). Together, these results indicate that AP is associated with both compositional shifts and alterations in the structure of the gut virome.

In parallel, bacterial community diversity was also evaluated. No significant difference was observed in the observed number of bacterial taxa between the AP and HC groups (*P* > 0.05; Fig. S1A). However, the Shannon diversity index was significantly reduced in AP patients compared with HC (*P* < 0.001; Fig. S1B), indicating decreased bacterial community evenness. Beta diversity analysis further revealed a significant separation in bacterial community composition between the two groups. PCoA based on Bray–Curtis dissimilarity demonstrated a clear compositional shift (R² = 0.0628, *P* = 0.001; Fig. S1C), while Jaccard-based PCoA yielded consistent results (R² = 0.0393, *P* = 0.001; Fig. S1D), indicating pronounced alterations in the overall structure of the gut bacteriome.

### Identification of gut virome features associated with AP

**Fig 2:**
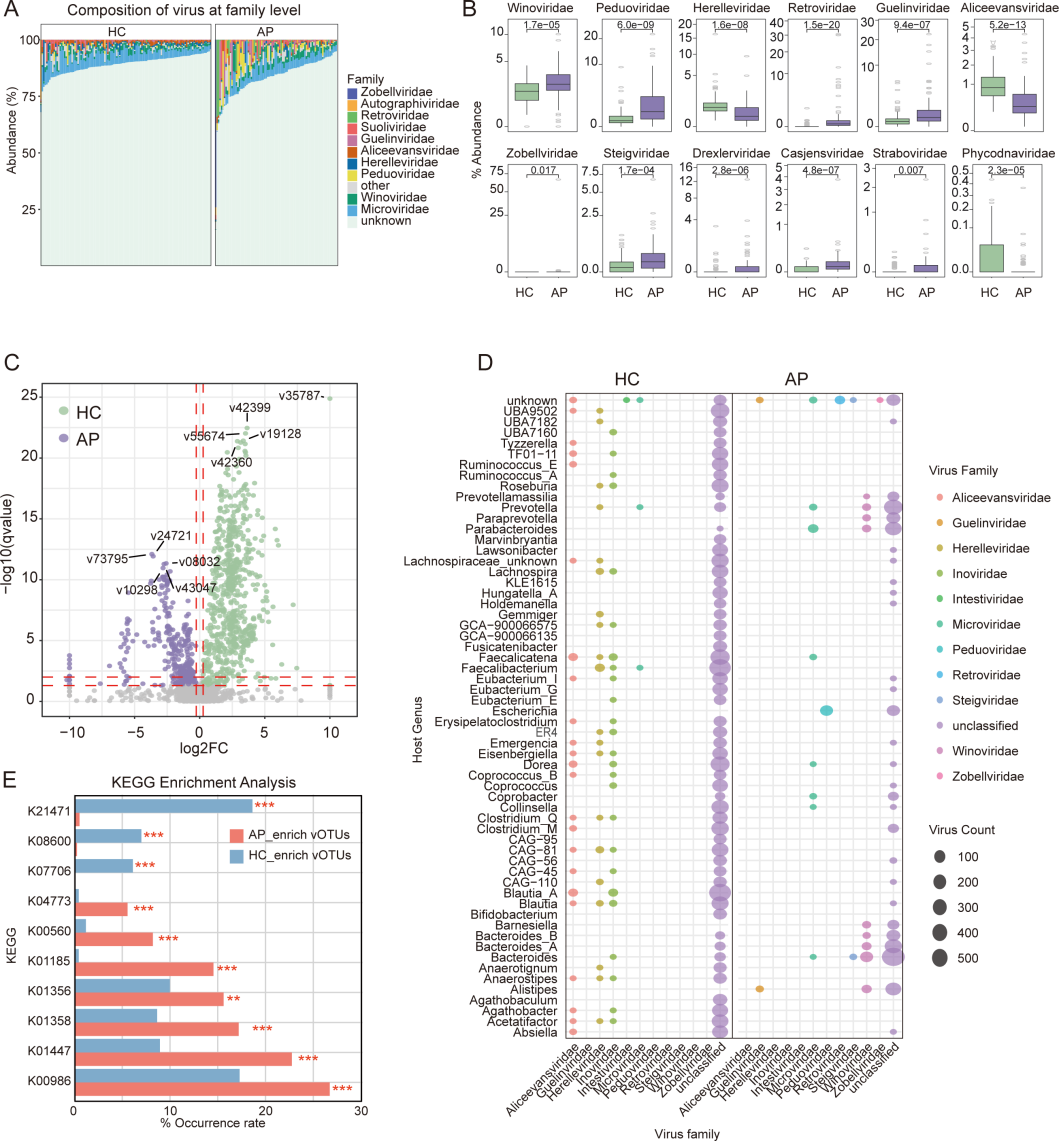
Viral taxonomic and host interaction signatures associated with AP. (**A**) Taxonomic composition of the gut virome at the viral family level (top 10 families). (**B**) Box plots showing differentially abundant viral families between AP and HC groups, identified by the Wilcoxon rank-sum test (*P* < 0.05). (**C**) Volcano plot displaying differential abundance of vOTUs between AP and HC groups. Purple dots indicate vOTUs significantly enriched in the AP group (fold change > 1.2; Benjamini–Hochberg-adjusted q < 0.05), green dots indicate vOTUs enriched in the HC group, and gray dots represent non-significant vOTUs. (**D**) Dot plots illustrating predicted bacterial host genera associated with vOTUs enriched in AP and HC groups. (**E**) Functional enrichment of AMGs encoded by vOTUs. Red bars represent KO frequencies among AP-enriched vOTUs, while blue bars indicate those enriched in HC-associated vOTUs.

Next, we investigated the taxonomic composition of the gut virome at the viral family level. Notably, 86.37% ± 8.08% of vOTUs could not be assigned to any known viral family, reflecting a substantial proportion of taxonomically unclassified viruses. Among the classified fraction, both AP patients and HCs were predominantly composed of *Microviridae* and *Winoviridae* (Fig. 2A). Notably, nine viral families—including *Peduoviridae*, *Guelinviridae*, *Winoviridae*, and *Retroviridae*—were significantly enriched in the AP group, while *Herelleviridae* and *Phycodnaviridae* were significantly depleted compared to the HC group (adj-*P* value < 0.05, FC > 1.2, Fig. 2B; Table S2). These results suggest that gut viral community is associated with the pathogenesis of AP.

Inter-group comparison at the vOTU level (fold change > 1.2, adj-*P* value < 0.05, relative abundance > 0.01%) revealed substantial differences in gut virome composition between the AP and HC groups (Fig. 2C; Table S3). A total of 1,049 differentially abundant vOTUs were identified, with 378 significantly enriched in the AP group and 671 enriched in HCs. MaAsLin2-based multivariable validation confirmed the majority of these findings, with >79% of differentially abundant vOTUs remaining significant and showing consistent directionality between AP and HCs (Table S3).

To explore potential host associations, we predicted vOTU-host linkages using two complementary sequence-based approaches: CRISPR spacer matching and homology-based alignment. The results showed that vOTUs enriched in the AP group were predominantly assigned to bacterial genera, such as *Parabacteroides*, *Escherichia*, and *Bacteroides*, while those enriched in the HC group were mainly linked to *Faecalicatena*, *Faecalibacterium*, and *Blautia_A* (Fig. 2D; Table S4).

To assess the functional potential of the gut virome, we profiled viral AMGs by annotating predicted viral proteins against the KEGG database, identifying 4,690 unique KOs across 1,049 vOTUs. Occurrence-based analysis revealed that 11 AMGs differed significantly in prevalence between the AP and HC groups (Fisher test, adj-*P* value < 0.01, occurrence rate > 5%; Fig. 2E; Table S5). Specifically, 542 AMG were more frequently encoded by AP-enriched vOTUs, including K00986 (RNA-directed DNA polymerase) and K01185 (lysozyme), whereas 599 AMG were more common among HC-enriched vOTUs, such as K21471 (peptidoglycan DL-endopeptidase CwlO), K08600 (sortase B), and K07706 (sensor histidine kinase). These AMG differences indicate distinct viral functional capacities linked to virus–bacterium interactions and potential host modulation across disease states, which may contribute to AP pathogenesis.

### Etiology and severity of AP are associated with gut microbial signatures

**Fig 3:**
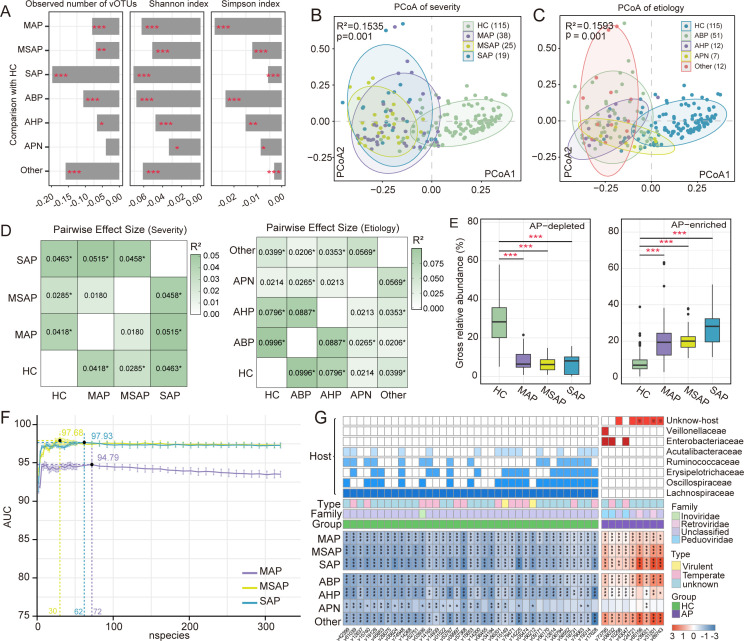
Gut virome signatures associated with AP etiology and severity. (**A**) Barplots comparing alpha diversity indices (species richness, Shannon index, and Simpson index) between healthy controls and AP subgroups stratified by disease severity and etiology. (**B and C**) PCoA based on Bray–Curtis dissimilarity, showing virome compositional differences across disease severity levels (**B**) and etiological subtypes. Each point represents a sample; ellipses indicate 95% confidence intervals. (**D**) PERMANOVA-based analysis of virome composition showing significant differences among AP subgroups categorized by severity and etiology. R² values represent the proportion of variation explained. (**E**) Boxplots showing the cumulative relative abundance of AP-depleted (left) and AP-enriched (right) vOTUs across severity levels (MAP → MSAP → SAP). (**F**) AUC performance curves of random forest models classifying AP severity subgroups based on varying numbers of features. (**G**) Heatmap showing the top 30 vOTUs most strongly associated with AP severity. Each column represents a vOTU, and each row represents a clinical subgroup of acute pancreatitis (MAP, MSAP, SAP). Colors indicate the relative abundance (log-transformed and normalized) of each vOTU across severity groups. vOTUs are annotated by viral family (if available), lifestyle, and predicted bacterial host taxonomy. *Adj-*P* value < 0.05, **adj-*P* value < 0.01, ***adj-*P* value < 0.001, ※ indicates eukaryotic viruses.

To further investigate how disease severity and etiology influence AP-associated virome features, we analyzed viral community differences across clinical subgroups. Alpha diversity analysis showed that there were no significant differences among AP subgroups stratified by etiology (Fig. S2A). However, compared with HCs, viral species richness, Shannon index, and Simpson index were significantly decreased across AP subgroups stratified by disease severity (Fig. 3A; Fig. S2B). In terms of overall community structure, PERMANOVA analysis demonstrated significant differences in virome composition associated with both AP severity (*P* = 0.001, *R²* = 0.1535; Fig. 3B) and etiology (*P* = 0.001, *R²* = 0.1593; Fig. 3C). Notably, substantial virome compositional shifts were also observed between AP subgroups themselves, particularly across the severity spectrum (Fig. 3D). Moreover, patients with APN exhibited a distinct gut virome profile compared to those with ABP or AHP etiologies.

In parallel, bacterial alpha diversity was further stratified by AP etiology and disease severity. Across etiological subgroups, the observed number of bacterial taxa was significantly reduced in AP patients compared with HCs (Fig. S2C). Consistently, the Shannon diversity index was significantly lower in multiple etiological subgroups, particularly in ABP and AHP groups, indicating reduced bacterial community evenness. Similarly, severity-based stratification revealed a progressive decline in bacterial alpha diversity with increasing disease severity (Fig. S2D). Both species richness and Shannon index were significantly reduced in MAP, MSAP, and SAP patients compared with HCs, with the lowest diversity observed in SAP patients. These results suggest that bacterial community diversity is broadly disrupted in AP and is further modulated by both disease etiology and severity.

We next examined the overall abundance patterns of AP-enriched and AP-depleted vOTUs across different severity stages. A clear gradient was observed along the MAP → MSAP → SAP continuum, with both groups of vOTUs exhibiting stepwise changes (Fig. 3E). Based on this trend, a total of 320 vOTUs demonstrated consistent severity-associated trajectories: 126 AP-enriched vOTUs increased in abundance with disease progression, while 194 AP-depleted vOTUs showed a progressive decline. Using these vOTUs, we constructed random forest models to classify patients by disease severity. Remarkably, these vOTU-based signatures achieved high predictive accuracy, with all AUC values exceeding 90% (Fig. 3F), highlighting a strong association between gut viral composition and the progression of acute pancreatitis. To further characterize these severity-associated virome signatures, we visualized the top 10 vOTUs that exhibited the most pronounced monotonic increases or decreases in relative abundance along the MAP–MSAP–SAP gradient (Fig. S3). These representative vOTUs displayed clear and consistent abundance trends across disease stages, reinforcing their close association with AP severity. Although their functional roles remain to be fully elucidated, the marked and progressive alterations of these viruses suggest that they may be potentially involved in the onset and progression of acute pancreatitis.

To further explore the functional and taxonomic relevance of these vOTUs, we analyzed the top 30 ranked features from three random forest models (Fig. 3G). Although many vOTUs lacked clear family-level annotations, reflecting current limitations in viral reference databases, several members of the family *Peduoviridae* were consistently enriched across different stages of AP, suggesting a potential involvement of this viral family in AP pathogenesis. At the host level, AP-depleted viruses were primarily associated with commensal bacterial families, such as *Lachnospiraceae*, *Erysipelotrichaceae*, *Oscillospiraceae*, *Acutalibacteraceae*, and *Ruminococcaceae*. In contrast, AP-enriched viruses were predominantly associated with *Enterobacteriaceae* and *Veillonellaceae*, with lysogenic viruses in this group predicted to infect *Enterobacteriaceae*—a bacterial family known to harbor numerous gut-associated pathobionts. This pattern may reflect one of the potential mechanisms through which the gut virome contributes to AP pathogenesis.

Finally, we identified a significant enrichment of eukaryote-infecting viruses among the AP-associated vOTUs. This suggests that, beyond classical virus–bacteria interactions, certain viral populations may influence AP progression through additional host-related mechanisms. Although further validation is required, these findings indicate that the virome may play a more complex and actively involved role in the pathogenesis of acute pancreatitis.

### Correlations among AP-associated gut viruses, bacteria, and clinical biochemical variables

**Fig 4:**
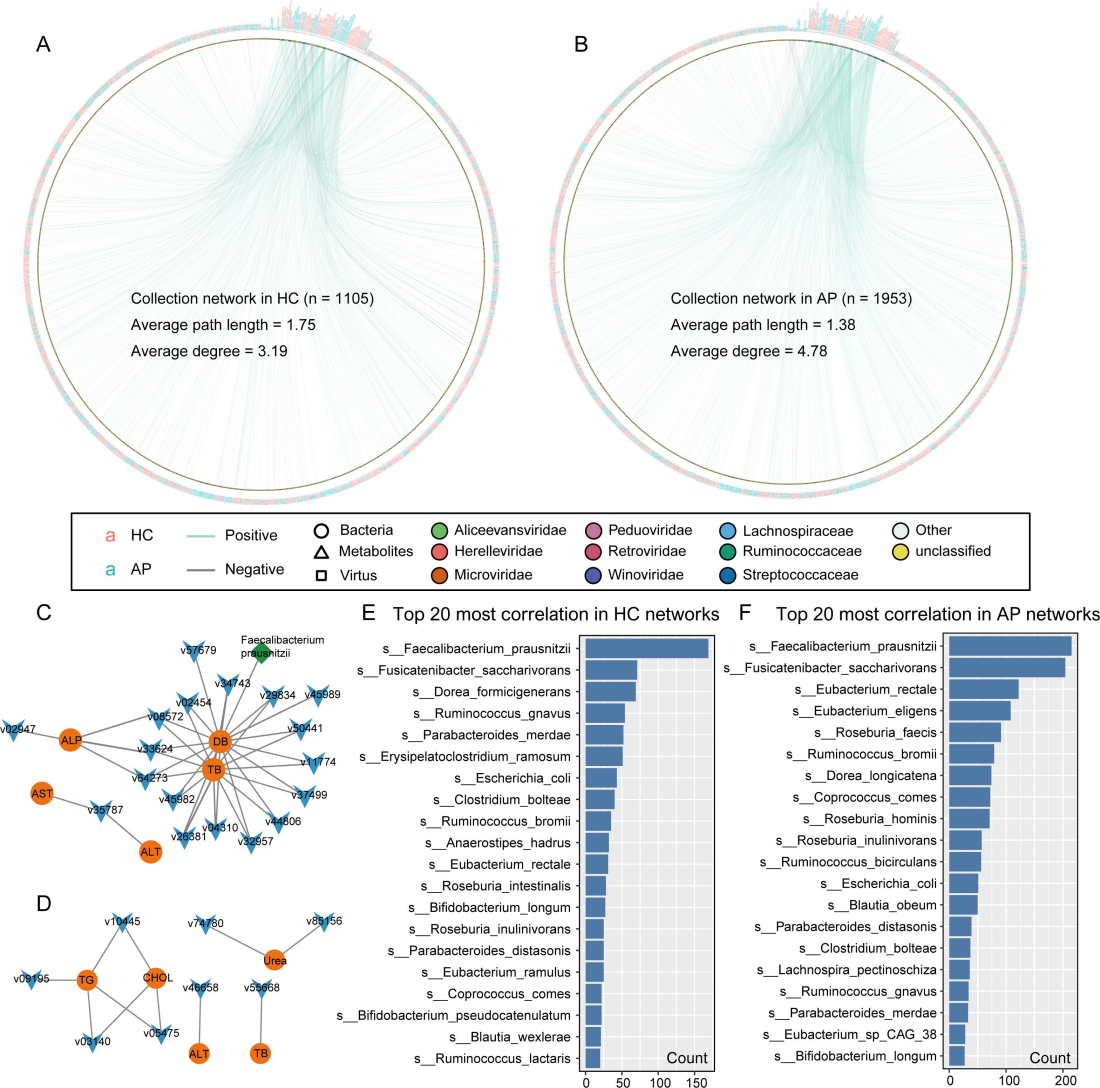
Cross-kingdom co-abundance networks in AP and HC groups. (**A, B**) Spearman correlation networks showing relationships among viruses (squares), bacteria (circles), and clinical variables (triangles) in HC (**A**) and AP (**B**) groups. Node colors represent viral or bacterial taxonomic families or clinical variables. Edges represent significant correlations (|ρ| > 0.4, q < 0.05): green for positive correlations (ρ > 0.4) and gray for negative correlations (ρ < 0.4). (**C** and **D**) Subnetworks showing representative virus-metabolite and bacterium-metabolite associations in HC (**C**) and AP (**D**). (**E and F**) Bar plots showing the top 20 microbial taxa with the highest degree (number of connections) in the HC (**E**) and AP (**F**) networks.

To explore potential host–virus interactions and their associations with clinical features, we conducted Spearman correlation analysis between 1,049 vOTUs, 68 bacterial taxa, and 15 clinical indicators. Using the same filtering criteria applied to viral species, we identified 68 AP-associated bacterial taxa that were significantly correlated (|ρ| > 0.4; adj-*P* value < 0.05) with the 1,049 vOTUs and 15 clinical parameters. These clinical parameters included key indicators of disease severity and organ function, encompassing inflammatory markers (white blood cell count and percentage of neutrophils [%NEUT]), markers of liver injury and cholestasis (alanine aminotransferase [ALT], aspartate aminotransferase [AST], gamma-glutamyl transferase [γ-GT], alkaline phosphatase [ALP], total bilirubin [TB], and direct bilirubin [DB]), as well as metabolic indicators, including cholesterol, triglycerides, and blood glucose. These correlations revealed distinct co-occurrence patterns between viruses, bacteria, and host clinical features in AP patients compared to HCs (Fig. 4A and B; Tables S6 and S7). Notably, the AP network exhibited a markedly higher density of virus–bacterium–metabolite interactions, along with a substantial increase in negative correlations between clinical metabolic indicators and microbial taxa. Specifically, the AP network contained 1,953 significant correlations, compared to only 1,105 in the HC network. Topological analysis further revealed that the AP-associated microbial-metabolite-virus network had a higher average degree (4.78 vs 3.19) and a shorter average path length (1.38 vs 1.75) than the HC network, indicating a more densely connected and topologically compact structure. These features suggest intensified biological interactions and more efficient communication within the microbial ecosystem in the disease state, potentially contributing to the propagation of dysbiosis and host metabolic disturbances in AP.

In particular, a large number of viral species and several bacterial groups, such as *Clostridium* spp., *Parabacteroides*, and *Erysipelatoclostridium ramosum*, were significantly negatively correlated with liver function and metabolic markers, including ALT, DB, Glu, r_GT, and ALP. (Fig. 4C and D). In contrast, the HC network showed far fewer such associations, with only sparse negative correlations observed between viral taxa and DB. Moreover, in the AP network, we observed numerous positive correlations between CHOL levels and both gut bacteria and viruses, suggesting that altered lipid metabolism may be closely associated with microbial activity in the disease state. Conversely, in the healthy group, only ALP and TB showed positive correlations with viruses, and no meaningful associations were observed with bacterial taxa.

Among the microbial taxa involved, *Fusicatenibacter saccharivorans, Eubacterium rectale, Eubacterium eligens*, and *Roseburia faecis* exhibited significantly increased network connectivity in the AP group (Fig. 4E and F). These commensal species—known for producing short-chain fatty acids—may serve as central nodes in the perturbed microbial network, either attempting to compensate for dysbiosis or reflecting increased ecological sensitivity under disease-associated stress. Their elevated network centrality suggests functional importance and potential involvement in the pathophysiology of AP.

Taken together, these findings highlight the complexity and clinical relevance of host–microbe–metabolite interactions in acute pancreatitis. The presence of distinct correlation structures in AP versus healthy networks suggests that specific microbes and metabolites may contribute to, or reflect, disease severity and progression.

### Diagnostic potential of enteric viruses and bacteria

**Fig 5:**
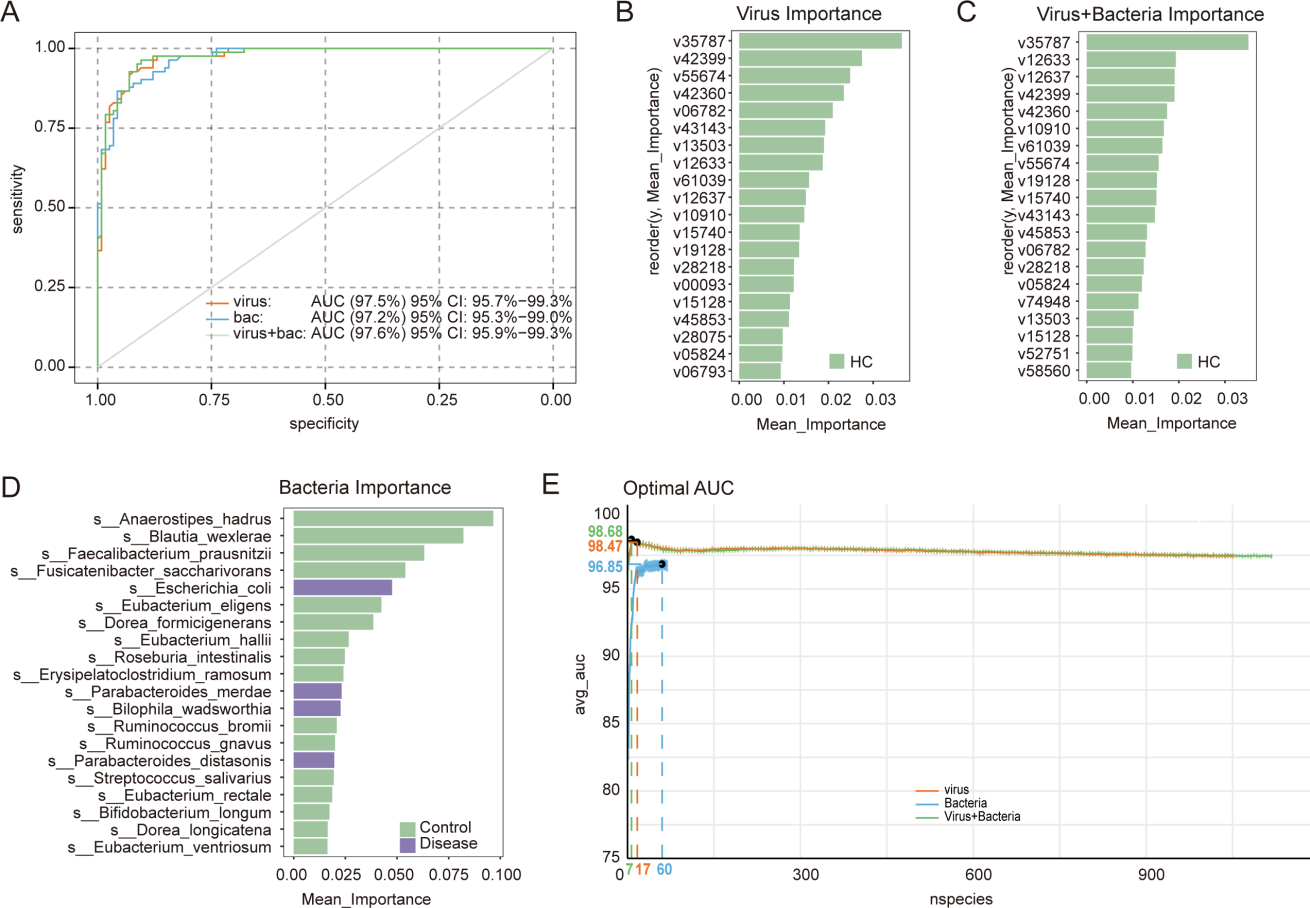
Diagnostic performance of gut microbial signatures for AP classification. (**A**) Receiver operating characteristic (ROC) curves showing the classification performance of Random Forest models trained using bacterial features, viral features, and combined bacterial-viral features. (**B–D**) Feature importance plots displaying the top 20 microbial predictors contributing to classification in the virus-only model (**B**), the combined virus-bacteria model (**C**), and the bacteria-only model. (**E**) AUC performance curves showing model accuracy across varying numbers of input features.

We evaluated the diagnostic performance of gut microbial signatures for AP classification using Random Forest models based on bacterial, viral, and combined bacterial-viral features. All models demonstrated high discriminative power, with AUCs of 97.2% (bacteria-only), 97.5% (virus-only), and 97.6% (combined model) (Fig. 5A). Importantly, viral features dominated the top 30 ranked predictors in the combined model, suggesting that the virome provides stronger diagnostic signals than the bacteriome (Fig. 5B through D).

To directly compare the diagnostic utility of viral and bacterial markers, we evaluated model performance across different numbers of selected features. The viral model consistently outperformed the bacterial model at every feature count and reached maximal accuracy using only seven viral features (Fig. 5E). In contrast, the bacterial model showed lower accuracy regardless of the number of markers included. These findings indicate that viral signatures not only possess strong diagnostic potential but surpass bacterial markers in predictive performance, highlighting the virome as a more sensitive and efficient biomarker source for AP classification.

## DISCUSSION

Recent studies have shown that gut virome alterations are highly disease-dependent. For example, a longitudinal IBD study using non-amplified viromes reported strong interpersonal variability and limited associations between the virome and disease status, suggesting that viral signals may be difficult to detect in conditions with high temporal fluctuation and substantial individual heterogeneity (29). In contrast, another large-scale IBD meta-analysis integrating multiple cohorts revealed robust and reproducible viral signatures across populations, indicating that virome-disease associations can emerge clearly under certain pathological contexts (30). Together, these findings highlight that the visibility of virome alterations is strongly shaped by disease characteristics, ecological stability, and inflammatory intensity. Given that acute pancreatitis is an abrupt-onset disease accompanied by profound microbial disruption and systemic inflammation, defining its virome landscape becomes particularly important (22). In this acute inflammatory setting, viral community shifts may be more pronounced, mechanistically meaningful, and clinically informative, underscoring the necessity and relevance of our AP-focused virome investigation.

In this study, we present a comprehensive characterization of the gut virome in AP, integrating viral diversity, taxonomic and functional composition, predicted host associations, ecological networks, and diagnostic modeling. By leveraging a large-scale genome catalog (cnGVC) and robust metagenomic analysis, we demonstrate that AP is accompanied by marked reductions in viral community evenness, significant compositional restructuring, and strong associations with both disease severity and etiology. These changes suggest that the gut virome is dynamically associated with AP pathogenesis and progression (31), and that the observed alterations are not merely passive consequences of the disease.

Although the total number of observed vOTUs did not differ significantly between AP patients and HCs, both Shannon and Simpson indices were significantly lower in AP, indicating a reduction in community evenness. Such a shift implies dominance by a subset of viral taxa, potentially reflecting decreased ecological resilience and increased vulnerability to perturbations. Similar trends have been reported for the bacterial microbiome in AP, where overgrowth of opportunistic species and depletion of beneficial commensals lead to diminished diversity and stability (18). Beta diversity analysis and PCoA revealed clear separation between AP and HC groups, underscoring a broad reorganization of the viral community rather than isolated taxon-level changes.

Taxonomic profiling at the viral family level revealed that both groups were dominated by *Microviridae* and *Winoviridae*, along with numerous unclassified viral families. However, significant differential abundance of specific viral families implies potential pathological associations with AP. AP-enriched families, including *Retroviridae* and *Peduoviridae*, likely contribute to disease progression via multiple mechanisms. For example, viruses of the *Retroviridae* family exist in various vertebrate hosts. Studies have shown that the *Retroviridae* family is associated with the physiology and pathology of many diseases (32–34). Although direct evidence linking *Retroviridae* to acute pancreatitis is currently limited, the enrichment of this viral family observed in our study indicates a potential association that warrants further investigation. In parallel, the expansion of phage families, such as *Peduoviridae* and *Casjensviridae,* may reflect underlying bacterial dysbiosis. Their increased lytic activity during dysbiosis may lead to a heightened release of bacterial products, potentially exacerbating local inflammation and contributing to intestinal barrier impairment (35, 36), thereby facilitating bacterial translocation along the gut-pancreas axis—a known trigger for secondary infections in AP (37).

A central observation is the selective enrichment of phages linked to dysbiosis-associated bacterial hosts (*Parabacteroides*, *Escherichia*, *Bacteroides*) and the depletion of phages associated with commensal SCFA producers (*Faecalicatena*, *Faecalibacterium*, *Blautia_A*). This host shift echoes patterns reported in gut dysbiosis during inflammatory bowel disease (22, 38, 39), where phage expansion can exacerbate bacterial turnover, disrupt metabolic stability, and amplify host inflammatory signaling. The enrichment of lysogenic phages infecting *Enterobacteriaceae* in AP is particularly noteworthy, as prophage induction under inflammatory stress has been shown to facilitate horizontal gene transfer and enhance bacterial virulence (39). Functional annotation reinforces this mechanistic view. AP-enriched viruses displayed a higher frequency of AMGs related to DNA polymerase activity and lytic functions (e.g., lysozyme), consistent with an enhanced genetic potential for viral replication and bacterial cell wall degradation (40, 41). In contrast, HC-enriched phages carried AMGs potentially involved in maintaining bacterial structural integrity and host-microbe homeostasis (42, 43). This functional divergence suggests that virome alterations in AP may shift the balance from stabilizing bacterium–phage interactions toward destabilizing, pro-inflammatory dynamics.

The monotonic shifts of 320 vOTUs across MAP → MSAP → SAP stages are reminiscent of bacterial gradients observed in AP severity. Specifically, enrichment of *Peduoviridae*, known to infect Enterobacteriaceae, aligns with evidence that Enterobacteriaceae expansion correlates with severity and poor prognosis in AP (44). Our random forest models based on these vOTUs achieved AUCs >0.90 for severity classification, underscoring their prognostic potential. Mechanistically, biliary obstruction in ABP may alter gut virome structure through bile acid flux changes and luminal pH shifts (45), while metabolic etiologies, such as APN, may act primarily via systemic inflammation and microcirculatory impairment (46).

Network analysis revealed that the AP microbial-viral-metabolite network was denser, more compact, and more highly connected than in HCs (average degree 4.78 vs 3.19; average path length 1.38 vs 1.75). Such compact networks can facilitate rapid propagation of perturbations across microbial and metabolic layers, potentially amplifying inflammatory cascades and accelerating metabolic dysregulation (47). The AP network showed more negative correlations between microbes and clinical indicators, such as ALT, DB, glucose, GGT, and ALP, and more positive correlations between total cholesterol and both bacterial and viral taxa, suggesting that altered lipid metabolism is closely tied to microbial activity in AP. Similar network densification has been observed in bacterial-metabolite networks during AP (48). Additionally, inverse correlations between viral abundance and TB/ALT levels in AP patients may reflect phage-mediated suppression of LPS-producing pathobionts, thereby limiting endotoxin release and downstream pro-inflammatory cytokine signaling (e.g., IL-6 and TNF-α) and alleviating hepatocellular injury and bilirubin dysmetabolism (49). In contrast, the positive associations observed in HCs are more consistent with a homeostatic host–microbe–virus equilibrium, in which viral and bacterial populations fluctuate in parallel with physiological variations in host metabolic indicators, without triggering inflammatory pathology (50–52). Together, these observations suggest a gut-liver regulatory axis in which virome–bacterium interactions influence hepatic metabolic homeostasis under both physiological and pathological conditions.

The observed performance of virus-based models highlights a strong association between the gut virome and AP. Virus-only models achieved AUCs comparable to, and in some cases exceeding, those of bacteria-only models, and a compact seven-virus signature maximized predictive performance. Combined models remained largely driven by viral predictors, underscoring the unique contribution of the virome beyond bacterial features. While the initial clinical diagnosis of AP is generally straightforward, early assessment of disease severity, etiological subtypes, and dynamic disease progression at hospital presentation remains challenging. In this context, lean viral panels may hold translational potential not as primary diagnostic tools, but rather for early risk stratification, severity assessment, and longitudinal monitoring of disease progression and host response throughout the clinical course.

This study has limitations. First, although rarefaction curves captured substantial diversity, neither depth nor sample size saturated low-abundance viruses, likely underestimating rare components. Second, host predictions and functional annotations for viral contigs remain constrained by database coverage and assembly contiguity; experimental host range validation and transcript-level functional assays will be critical next steps. Third, cross-sectional sampling limits causal inference. Longitudinal studies through AP onset, peak, and recovery—ideally with medication, diet, and antibiotic metadata—are needed to disentangle drivers from consequences. Finally, while our models generalize within this cohort, external validation across centers and in larger, prospective studies is required to assess their potential for clinical translation.

### Conclusion

This study provides a comprehensive characterization of the gut virome in acute pancreatitis, revealing marked reductions in viral community evenness, large-scale compositional restructuring, and distinct functional shifts that are closely linked to both disease severity and etiology. AP-associated virome alterations were typified by expansion of phages targeting opportunistic or pro-inflammatory bacterial hosts and depletion of phages linked to commensal SCFA producers, accompanied by functional enrichment in genes related to viral replication and bacterial lysis. These taxonomic and functional changes were reflected in denser, more compact microbial-viral-metabolite networks and in specific associations with hepatobiliary and metabolic markers, suggesting an active role of the virome in AP pathogenesis through modulation of host–microbe interactions and metabolic homeostasis. Virome-based signatures demonstrated high diagnostic and prognostic potential, with a minimal viral panel achieving robust performance, underscoring their promise as non-invasive biomarkers for AP detection and severity stratification. Together, our findings highlight the importance of incorporating the gut virome into the current bacteriocentric framework of AP research and lay the groundwork for future mechanistic and translational studies aimed at virome-targeted diagnostics and therapeutics.

## Data Availability

The metabolomics data sets reported in this study, the analysis code supporting this study, and the completed STORMS Checklist are publicly available at https://github.com/xxx265/AP_virome.

## SUPPLEMENTAL MATERIAL

Supplemental figures msystems.01400-25-s0001.pdf Fig. S1 to S3.

Supplemental tables msystems.01400-25-s0002.xlsx Tables S1 to S7.

ASM does not own the copyrights to Supplemental Material that may be linked to, or accessed through, an article. The authors have granted ASM a non-exclusive, world-wide license to publish the Supplemental Material files. Please contact the corresponding author directly for reuse.
