# Supplementary material for: Gut virome and metabolic associations in patients with acute pancreatitis
Source: mSystems. 2026 Mar 6;11(3):e01400-25. doi: 10.1128/msystems.01400-25 (PMC13011395; doi:10.1128/msystems.01400-25)

Supplementary Figure 1. Diversity and compositional profiles of the gut bacteriome in acute pancreatitis (AP) patients and healthy controls (HC). **(A–B)** Boxplots showing α-diversity metrics of the gut bacteriome, including the observed number of bacterial taxa (A) and the Shannon index (B), in HC and AP groups. **(C)** Principal coordinates analysis (PCoA) of bacterial community composition based on Bray–Curtis dissimilarity. **(D)** PCoA of bacterial community composition based on Jaccard distance. Each point represents one sample, and ellipses indicate the 95% confidence intervals for each group.

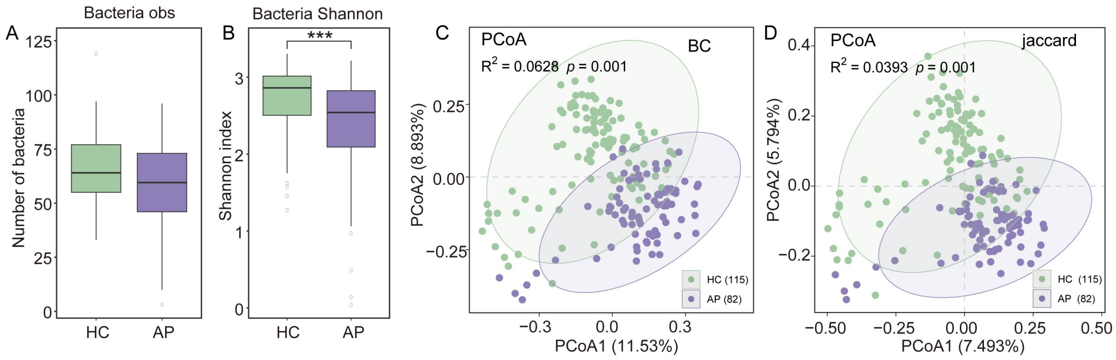

Supplementary Figure 2. Alpha-diversity of the gut virome and bacteriome across acute pancreatitis subgroups. **(A–B)** Boxplots showing viral alpha-diversity metrics, including the observed number of viral operational taxonomic units (vOTUs) and the Shannon index, across groups stratified by etiology (A; HC, ABP, AHP, APN, and Other) and disease severity (B; HC, MAP, MSAP, and SAP). **(C–D)** Boxplots showing bacterial alpha-diversity metrics, including the observed number of bacterial taxa and the Shannon index, across the same etiology-based (C) and severity-based (D) subgroup classifications. HC, healthy controls; ABP, acute biliary pancreatitis; AHP, acute hyperlipidemic pancreatitis; APN, acute necrotizing pancreatitis; MAP, mild acute pancreatitis; MSAP, moderately severe acute pancreatitis; SAP, severe acute pancreatitis.

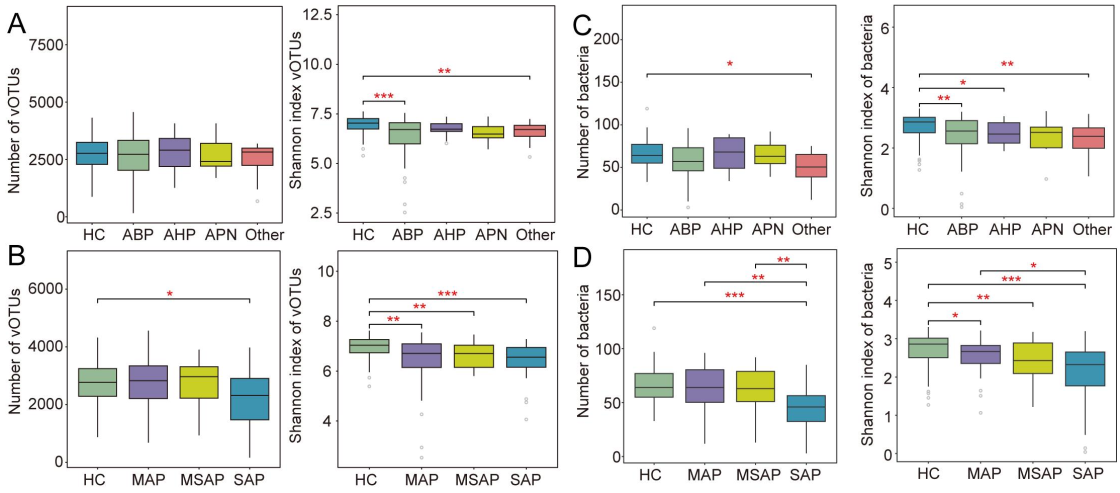

Supplementary Figure 3. Relative abundance of representative vOTUs across acute pancreatitis severity groups. **(A)** Boxplots showing the relative abundance of the top 10 vOTUs selected based on increasing patterns across disease severity groups. **(B)** Boxplots showing the relative abundance of the top 10 vOTUs selected based on decreasing patterns across disease severity groups. Samples are grouped by disease severity, including healthy controls (HC), mild acute pancreatitis (MAP), moderately severe acute pancreatitis (MSAP), and severe acute pancreatitis (SAP). Each panel represents one vOTU, and relative abundance is shown as a percentage.

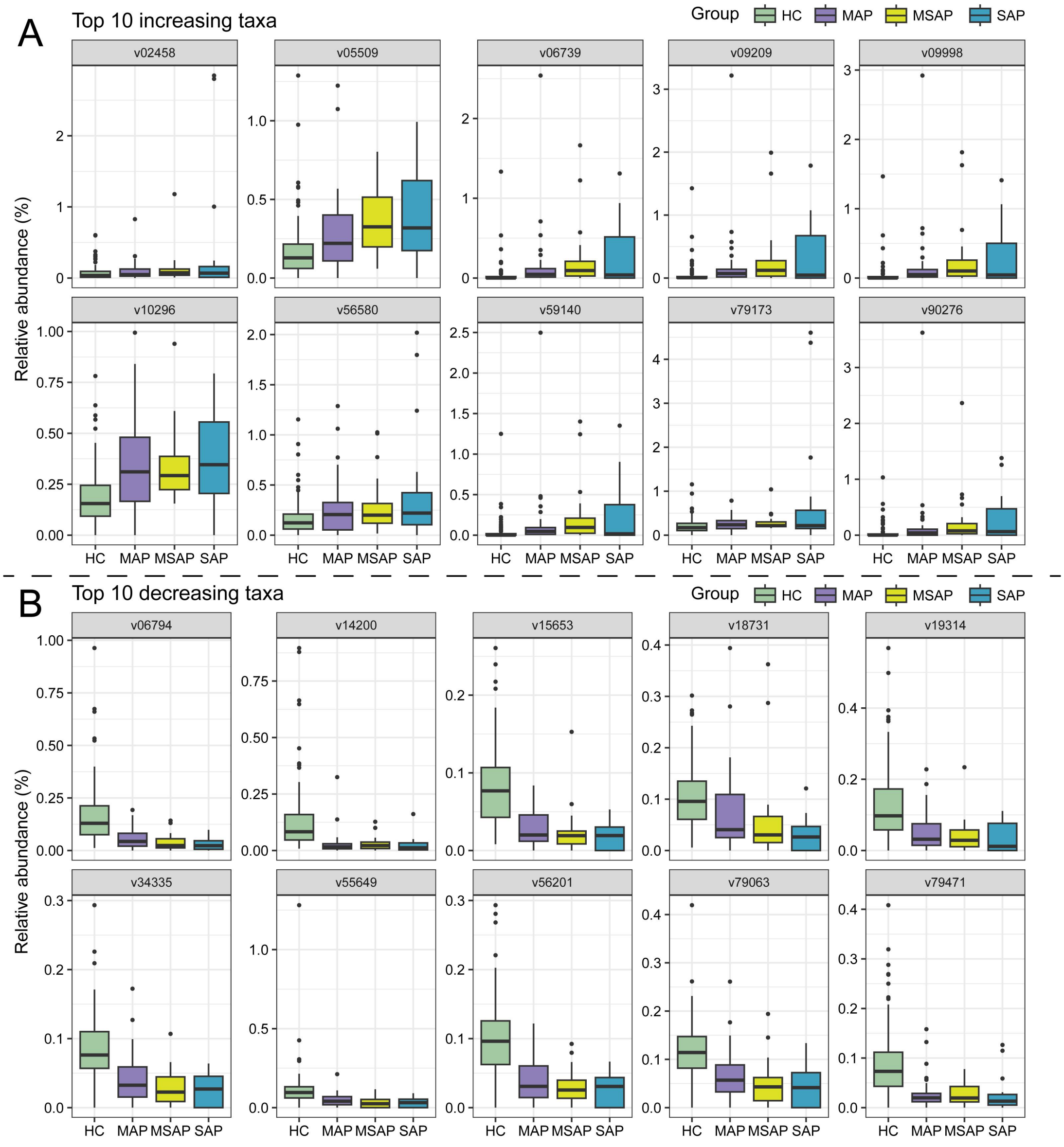

Supplement: Supplemental figures — Fig. S1 to S3. [file msystems.01400-25-s0001.pdf]
